# Effect of moderate-intensity exercise bouts lasting <10 minutes on body composition in sedentary Kenyan adults aged ≥50 years

**DOI:** 10.1136/bmjsem-2018-000403

**Published:** 2018-10-01

**Authors:** Karani Magutah, Nilesh B Patel, Kihumbu Thairu

**Affiliations:** 1 Department of Medical Physiology, Moi University, Eldoret, Kenya; 2 Department of Medical Physiology, University of Nairobi, Nairobi, Kenya

**Keywords:** cardio-respiratory fitness, sedentary, exercise bouts, body composition

## Abstract

**Background:**

Sedentary lifestyles and related morbidities are rising among adults despite existing exercise recommendations. Appealing exercise regimes yielding similar/better body composition should be sought.

**Objective:**

We investigated the effect of moderate-intensity exercise bouts of <10 min on body composition in previously sedentary adults.

**Methods:**

This unblinded study enrolled 53 healthy sedentary volunteers aged ≥50 years, randomised into one of two gender-balanced exercise interventions: (1) male and (2) female short-duration bouts (M_S_, n=14; F_S_, n = 13), and (3) male and (4) female long-duration bouts (M_L_, n=13; F_L_, n=13). Short-duration bouts entailed 5–10 min of jogging thrice daily; long-duration bouts, 30–60 min 3–5 days weekly. Body composition was determined at recruitment and 8-weekly thereafter, for 24 weeks.

**Results:**

At baseline, 14.3% of M_S_, 38.5% of M_L_, 92.3% of F_S_ and 69.2% of F_L_ were obese, dropping to 7.1%, 15.4%, 61.5% and 30.8%, respectively. For waist:height ratio, 64.3 % of M_S_, 76.9% of M_L_, 100% of F_S_ and 84.6.3% of F_L_ had ratios >0.5, dropping to 42.9%, 30.8%, 92.9% and 26.2%, respectively. While baseline M_S_ and M_L_ waist:hip ratio (WHR) ≥0.9 were 64.3% and 69.2%, respectively, they correspondingly dropped to 23.1% and 21.4%. The F_S_ and F_L_ with WHR ≥0.85 dropped from 46.2% to 15.4% and from 30.8% to 7.7%, respectively. Body composition variables improved for both sexes (all p <0.05) and mean change between exercise regimes was comparable for both sexes.

**Conclusion:**

In equal cumulative times, moderate-intensity exercise bouts lasting <10 min are comparable with current 30–60 min bouts in body composition modification for adults of ≥50 years.

**What are the new findings?**

- Moderate-intensity exercise bouts of <10 min have favourable effects on changing body composition in sedentary adults from either sex.
- There is no difference in the magnitude of body composition change from different exercise prescriptions as long as cumulative exercise durations are equal.

**How might it impact on clinical practice in the near future?**

- Individuals aged above 50 years could benefit from a review of current exercise recommendations.
- More appealing exercise prescription options for individuals at risk of the metabolic syndrome triad will make it easier to control inactivity-related non-communicable diseases.

## Introduction

Sedentary lifestyles and related morbidities are on the rise, and physical inactivity currently is the number four cause of death worldwide.^1 2^ Globally, 51%–79% of adults are physically inactive and do not meet their exercise recommendations.^2 3^ This trend has also been shown in sub-Saharan Africa, and physical inactivity has been identified as a major risk factor in the now increasing burden of lifestyle diseases here.^4 5^ In Eldoret, Kenya, site of the current study, 82% of adults are inactive.^6^ The rapid urbanisation and increasing sedentary lifestyles associated with ‘*the haves*’ in Kenya and indeed the larger sub-Saharan Africa today are thought to be contributory.^7^ This further compounds the lack of exercise time associated with daily engagements among the older populace that is variously also age-compromised for sustained exercise participation. This may be associated with the observed epidemiological transition where cardiovascular disease is on the rise among adults.^8–14^ Unchecked, this inactivity may lead to longer-term poor health effect on individuals and undermine the healthcare systems already overwhelmed by communicable diseases.^9–11 13 15–17^

Recommendations of ways to tackle such physical inactivity are in existence. Evidence that participation in exercise or physical activity improves body composition is plenty, although data among the elderly are still scanty.^2 18 19^ Available exercise and physical activity guidelines are, however, inadequately followed and exercise recommendations are not being achieved in adults.^2 3 6^ Adherence to exercise has been poor and debate on the best exercise regime that could help achieve this has not been settled for a few decades now.^2 20–26^

Debate on the value and effectiveness of shorter exercise bouts on body composition is unsettled. Even where studies have focused on the benefits of accumulated short bouts of moderate-intensity exercise, benefits have not been congruent.^27 28^ Further, observation that 30 min bouts yield poor adherence calls for further studies on the value of the shorter (but with cumulative time equalling the existing guidelines) bouts in body composition improvement. To the best of our knowledge, there has been minimal studies on the value of exercise regimes of bouts lasting <10 min in older individuals. Where some exist, they followed their participants for a short period (8 weeks) failing to answer the question of the long-term benefits.^24–26^ The current study compared body composition indicators among sedentary adults following different exercise regimes performed over a 24-week period to identify any distinct differences in the regimes.

## Materials and methods

We studied healthy sedentary adults aged at least 50 years (men=27; women=26) and residents of Eldoret town, Kenya. They volunteered in response to a local print advertisement. Using the WHO Global Physical Activity Questionnaire Sedentariness, sedentary individuals were those with <600 weekly metabolic equivalent (MET)-minutes of exercise. Further, only volunteers devoid of existing physical injuries and reported cardiovascular disease or treatment were included. All measurements were performed by the same researcher throughout for consistency and reproducibility.

The study was unblinded. Participants from either sex were randomly allocated into two groups per sex based on either of the two exercise prescriptions to be administered. This yielded four subgroups: (1) male short-duration bouts of exercise (M_S_), (2) female short-duration bouts of exercise (F_S_), (3) male long-duration bouts of exercise (M_L_) and (4) female long-duration bouts of exercise (F_L_). Those in the short-duration bouts of exercise, the experimental group, engaged in three daily bouts of 5–10 min of moderate-intensity jogging. Those in the long-duration bouts, the control group, involved in 30–60 min jogging bouts for 3–5 days weekly. Participants kept a record of exercise, which was objectively verified on select days using Polar Wearlink ActITrainer accelerometers (Actigraph, Pensacola, Florida, USA), monitors that detect activity and motion changes. These data were analysed weekly for confirmation that participants in the different exercise regimes had comparable MET-minutes.

The same researcher collected data from all the participants at recruitment stage and then at 8th, 16th and 24th week of exercise involvement. For each participant, height and weight without shoes but on light clothing were measured using a stadiometer and a mechanical scale (CAMRY Mechanical scale, BR9012, Shanghai, China). Waist and hip circumferences were measured with a tape measure. Skin-fold measures were taken using callipers (Harpenden Skinfold Callipers; BATY International, England). This was done from three different body sites: chest, abdomen and thigh for men; and the triceps, suprailiac and thigh for women. From these measurements, Body Mass Index (BMI), waist:hip ratio (WHR) and waist:height ratio (WHtR) were calculated. The sum of the three skin-fold measurements were used to determine body density using published generalised equations, while the fat percentage was computed from body densities using Brozek formulae.^29–32^

Data were analysed with STATA V.13. We used summary statistics and t-test functions, and outputs were in means and SD. Analysis was done at univariate, bivariate and multivariate levels based on sex and exercise regime adopted. Paired t-tests were conducted for exercise regime groups for each sex. Linear regressions controlling for the various predictor variables and potential confounders were further performed for the body composition outcome variables. Comparisons were evaluated at a set p value ≤0.05 for significance.

## Results

At the start point of the study, male participants in the experimental arm (n=14) were aged 55.0±5.6 years while those in the control group (n=13) were aged 55.2±3.0 years. Experimental arm women (n=13) were aged 53.9±2.6 years with their control group counterparts (n=13) at 53.9±3.5 years. Sixty-four per cent (64.3%) of M_S_ and 76.9% of M_L_, and 100% of F_S_ and 76.9% of F_L_ had acquired tertiary level of education. The rest had secondary-level training. All participants in the two experimental arms completed the 24-week exercise regime. In the control group, 61.5% of M_L_ and 76.9% of F_L_ completed the protocol. For BMI, 14.3% of M_S_, 38.5 of M_L_, 92.3% of F_S_ and 69.2% of F_L_ started as obese. Similarly, for WHtR, 64.3% of M_S_, 76.9% of M_L_, 100% of F_S_ and 84.6.3% of F_L_ had ratios >0.5. The M_S_ group with baseline WHR ≥0.9 were 64.3%, with M_L_ at 69.2%; the F_S_ whose baseline WHR was ≥0.85 were 46.2% and their F_L_ counterparts were 30.8%.

Mean values for these variables are presented in table 1.

**Table 1:** Baseline demographic and clinical characteristics of the participants

	M_S_	M_L_	F_S_	F_L_
Age (years)	55.0±5.6	55.2±3.0	53.9±2.6	53.9±3.5
BMI (kg/m^2)^	25.8±4.0	28.6±4.8	33.3±4.8	32.0±5.4
WHtR	0.52±0.07	0.56±0.08	0.61±0.05	0.57±0.08
WHR	0.93±0.06	0.96±0.07	0.82±0.10	0.84±0.09
Sum of 3 skin folds (mm)	62.2±27.75	78.9±31.2	120.3±20.6	109.6±23.81
Body density	1.05±0.02	1.04±0.02	1.01±0.01	1.01±0.01
Fat %	20.7±7.3	24.9±7.9	39.8±3.6	37.4±5.1

Data presented as mean±SD.

BMI, Body Mass Index; FL, female long-duration bouts of exercise; FS, female short-duration bouts of exercise; ML, male long-duration bouts of exercise; MS, male short-duration bouts of exercise; WHR, waist:hip ratio; WHtR, waist:height ratio.

At the end of the 24-week follow-up, M_S_, who had started as obese, reduced to 7.1% compared with a drop to 15.4% for M_L_. Similarly, obese F_S_ dropped to 61.5% versus a drop to 30.8% for F_L_. The percentage M_S_ whose WHtR was above 0.5 dropped to 42.9 compared with a drop to 30.8% for M_L_. For F_S_, it dropped to 92.3% versus a drop to 26.2% for F_L_. The M_S_ group whose baseline WHR was ≥0.9 dropped to 23.1% while M_L_ dropped to 21.4%, and for the F_S_ participants whose baseline WHR was ≥0.85, it dropped to 15.4% with the F_L_ counterparts dropping to 7.7%.

**Figure 1:**
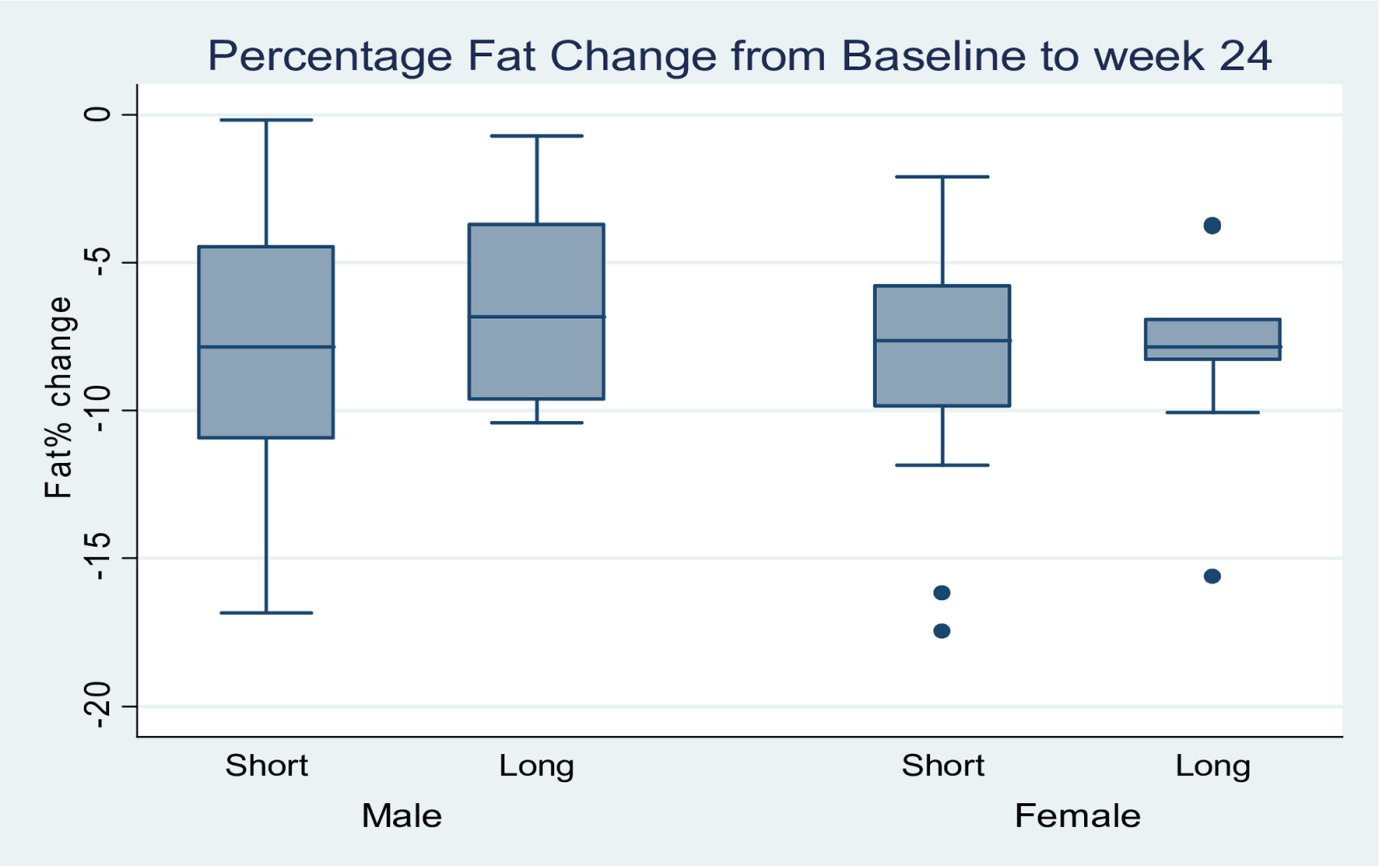
Percentage fat change between week 0 (baseline) and week 24 (endpoint).

Generally, all variables associated with body composition were found to decrease in measured values for both sexes. There were significant drops in body weight, BMI, WHtR, WHR and percentage body fat (all p<0.05). However, the mean change difference for each variable was not different between the two exercise regimes for both sexes as tabulated for the overall change from baseline values in table 2, and shown by the p values derived from the comparison of the mean change difference between the regimes. On controlling for sex, table 3 presents a summary of linear regressions for these variables. The change in percentage body fat for the two sexes and groups showed a similar drop as plotted in figure 1.

**Table 2:** Body composition measurements

Variable	Group	Baseline	Week 8	Week 16	Week 24	Mean change (week 24–week 0)	P values
Men							
Weight	Short	76.4±14.7	75.2±14.5	74.9±14.0	72.3±14.2	−4.07±5.8	0.74
	Long	82.8±16.2	82.3±15.8	81.4±15.3	79.4±15.8	−3.31±3.0	
BMI	Short	25.8±4.0	25.4±4.0	25.3±3.8	24.4±4.0	−1.37±2.0	0.75
	Long	28.2±5.0	27.9±4.6	27.7±4.7	27.0±4.1	−1.13±1.01	
WHtR	Short	0.52±0.07	0.51±0.06	0.50±0.06	0.49±0.05	−0.03±0.02	0.63
	Long	0.55±0.08	0.54±0.09	0.53±0.08	0.51±0.07	−0.04±0.02	
WHR	Short	0.93±0.06	0.92±0.06	0.90±0.05	0.87±0.05	−0.05±0.04	0.44
	Long	0.96±0.08	0.94±0.08	0.91±0.08	0.89±0.07	−0.06±0.03	
Fat %	Short	20.7±7.3	15.6±5.9	14.2±5.5	12.4±4.8	−8.32±5.0	0.36
	Long	21.6±8.5	19.3±7.5	17.3±6.3	15.2±5.8	−6.43±3.8	
Women							
Weight	Short	85.3±13.7	84.2±13.9	83.0±14.1	81.7±13.2	−3.61±1.54	0.22
	Long	78.1±11.0	75.0±9.6	73.9±9.6	72.5±9.9	−5.65±5.53	
BMI	Short	33.3±4.8	32.9±4.8	32.4±4.9	31.9±4.6	−1.41±0.60	0.25
	Long	30.5±5.1	29.3±4.6	28.9±4.9	28.4±5.1	−2.15±2.13	
WHtR	Short	0.605±.049	0.573±.054	0.565±.049	0.557±.050	−0.05±0.02	0.21
	Long	0.55±.085	0.534±.081	0.527±.085	0.518±.082	−0.04±0.02	
WHR	Short	0.822±.095	0.822±.065	0.811±.066	0.801±.066	−0.02±0.05	0.84
	Long	0.803±.078	0.794±.085	0.783±.081	0.785±.079	−0.02±0.04	
Fat %	Short	39.8±3.6	35.0±5.1	32.3±5.3	31.1±5.6	−8.69±4.41	0.67
	Long	35.8±4.4	30.8±4.6	29.3±4.9	27.8±4.9	−7.96±3.34	

Values are means±SD.

BMI, Body Mass Index; WHR, waist:hip ratio; WHtR, waist:height ratio.

**Table 3:** Linear regressions for body composition (controlling for sex)

Mean change, long	Coefficient	SE	P value>|t|	95% CI
Weight	−0.71	1.35	0.60	−3.44 to 2.02
BMI	−0.27	0.49	0.58	−1.25 to 0.71
WHtR	0.004	0.01	0.53	0.01 to 0.09
WHR	−0.004	0.01	0.75	0.03 to 0.02
Fat%	1.28	1.29	0.33	1.33 to 3.89

BMI, Body Mass IndexWHR, waist:hip ratio; WHtR, waist:height ratio.

## Discussion

### Body composition at start point

Men were overweight while women were obese (class I) at recruitment. Such BMI classes are associated with unfavourable health outcomes.^33–35^ The high baseline BMI of our participants suggests that they were at an increased risk for cardiovascular disease and, further, other pathologies associated with the metabolic syndrome. A few studies, however, propose that values within the 23–33 kg/m^2^ range have lower mortality risks among the elderly, so that the higher BMI values as observed in the current study may actually be associated with better cardiovascular disease epidemiology in this cohort.^36–39^ Based on this, women in the current study are especially safer.

Recently, studies have suggested that WHtR is superior to BMI in assessing risks associated with metabolic syndrome pathologies among different races and ages.^40–45^ Using the proposed WHtR cut-off of 0.5, participants in the current study were at cardiovascular disease risk at recruitment.^42–44 46^ In fact, considering proposals this ratio is superior to BMI in evaluating health risks and outcomes among apparently healthy populations as it particularly considers fat distribution, participants in this study had a considerably high risk for metabolic syndrome.^40 41 47^

A comparison of the baseline WHR against WHO cut-off guidelines of ≥0.90 (men) and ≥0. 85 (women) associated with substantially increased metabolic complications showed four in every five men and two in every five women had central/abdominal obesity with mean value above their respective WHO cut-offs.^48^ This indicates that at the start point, men in this study had a relatively higher risk for metabolic syndrome and other health problems, and bore a higher mortality risk.^35^ They had twice the risk portrayed by their female counterparts based on WHR alone.

Body composition reference data for the elderly African population with which comparison of the present study would be done are unavailable. While data abound elsewhere, these are based on non-African populations, and comparison may be affected by racial differences. Men and women in the current study had baseline percentage fat mass comparable with those found in an Indian population, although that study covered a wider age range.^49^ The high baseline percentage body fat for the women in the current study, making well over one-third of their body mass, and with a concurrent lower body density, is consistent with the baseline blood chemistry findings from another of our study on the same sample where the women had higher total cholesterol (TC) values.^50^ Based on Jackson and Pollock equations, men in this study had average baseline body fat, but their female counterparts were obese at the start point.^29–31^

These baseline findings indicated a potentially poor future health outcome for this cohort as such clinical features have been shown to get worse with advancing age and adoption of sedentary lifestyles, a finding consistent with other measures associated with cardiovascular disease independently and, broadly, the conglomeration of metabolic syndrome pathologies in the East-African region.^51–53^ Our exercise intervention and follow-up investigation improved these health parameters for the participants.

### Changes in body composition

Previous works have shown that 6–12 weeks of exercise is sufficient to cause body composition changes across all ages.^54–56^ Sustained exercise involvement is necessary to maintain any such gains, which is why the current study measurements were done 8-weekly for a follow-up period that allowed at least four measurements. Over the 24 weeks, all values on variables associated with body composition decreased in a similar manner for both men and women in the two exercise regimes. Although this drop was not to the recommended levels in some variables, it was significant nevertheless, and there was no demonstrable difference in the magnitude of change between regimes.

Given that BMI drop among the elderly to normal values as provided by the international WHO classification is a huge challenge, the percentage drop for our participants with BMI >24.9 kg/m^2^ was a significant achievement.^33^ Short bouts’ regime halved the percentage of obese men with the longer-bouts’ regime dropping the same by one-third. The longer bouts for the women had a marginally higher proportion of the drop for the obese when compared with the shorter. The finding, however, still resonates with a recent suggestion that shorter-frequent exercise bouts are efficient in weight management in obese and overweight women.^28^ Effectively, the reduction in the BMI for both men and women in the short bouts’ regime was similar to that observed in their longer bouts’ counterparts, with the comparison of the mean change for different regimes within each sex showing no significant difference over the 24 weeks.

Lower values of WHtR are associated with lesser cardiovascular disease risk and indeed other pathologies related to metabolic syndrome.^42 43^ In this study, there was a drop in the percentage of both men and women on short bouts whose WHtR remained >0.5 cut-off at the end of the 24-week follow-up. Similarly, the mean values for those whose ratios remained >0.5 dropped. Regardless of the exercise regime and whether or not participants attained a mean value <0.5, however, all participants in the current study portrayed a drop in their WHtR. While this drop was significant from the baseline to the endpoint, a similar trend of no demonstrable difference in the rate of the change based on the exercise regime was noticed. No previous work we are aware of has compared the effect of intermittent and traditional regimes with WHtR in such a follow-up as our current work in this regard. It is safe to argue that the two exercise regimes have similar modification of central obesity and body composition at large given that no difference was noticed in this aspect for the two regimes on controlling for sex.

A similar picture was observed for WHR. There was a more than half reduction in the percentage of both men and women whose WHR remained above the WHO cut-off of ≥0.9 and ≥0.85, respectively, in both the short and the long bouts’ regimes of exercise. The difference between the two bout types was, however, indistinct.^48^ For participants whose values remained above the WHO threshold, short bouts were found to nonetheless decrease the absolute values in a similar manner as the long bouts. Thus, no difference was demonstrable in the manner the two exercise regimes affected WHR. The short bouts were as effective as the long ones in reducing abdominal obesity and therefore the risk associated with high WHR values.^35^ On controlling for sex, no differences would yet be found between the short and the long regimes in this regard, depicting their similarity in effect.

Participants in this study had a drop in the percentage body fat, regardless of the exercise regime adopted. However, only the men had a drop to within their recommended levels based on the ideal body fat percentage with widest reference.^30^ The women had a significant drop as well regardless of their regime of exercise but could not attain their recommended ranges.^29^ This could be explained by fact that at the baseline, they had a higher baseline percentage body fat so that although there was a drop, only a longer follow-up period would have helped attain the recommended ideal levels. By a direct comparison of the two exercise regimes, the shorter bouts had a slightly although statistically insignificant advantage by causing an absolute higher mean drop of the fat percentage, in both men and women. Recent works on this area have found bouts lasting <10 min to be equally effective in lowering body fat in men and women when cumulative intermittent bouts are considered.^28 57^ That our study found no difference between the two exercise regimes on controlling for sex in this variable further shows that the short bouts’ regime of moderate-intensity exercise is at least as good as the traditional longer bouts in the regulation of body fat across sexes.

### Limitations

Generalisability of our findings may be affected by participants having been volunteers following a local print advert. Further, blinding was not done, and some lifestyle factors such as diet that would affect physical activity and cardiorespiratory fitness were not tracked given the logistical follow-up difficulties with this lengthy home-based trial, both of which may have confounded our results. That we used activity monitors on select days to verify the individually filled exercise logs may also have affected our results.

## Conclusions

As long as the aggregated exercise time and intensity be equivalent, bouts lasting up to 10 min are as effective as the traditional longer bouts of 30–60 min in modification of body composition among sedentary Kenyan adults of 50 years and above.
